# Loneliness and Its Associations With Personality Functioning: Evidence From Longitudinal Inpatient Psychotherapy Programs in Germany

**DOI:** 10.1111/nyas.70215

**Published:** 2026-02-04

**Authors:** Julia I. Kunz, Barbara B. Barton, Niklas Wolfrum, Johannes Wolf, Katharina Merz, Richard Musil, Stephan Goerigk, Andrea Jobst, Katja Bertsch, Frank Padberg, Matthias A. Reinhard

**Affiliations:** ^1^ Department of Psychiatry and Psychotherapy LMU University Hospital LMU Munich Munich Germany; ^2^ DZPG (German Center for Mental Health) Partner Site Munich‐Augsburg Munich Germany; ^3^ Oberberg Fachklinik Bad Tölz Bad Tölz Germany; ^4^ Charlotte Fresenius Hochschule Munich Munich Germany; ^5^ Department of Clinical Psychology and Psychotherapy Julius‐Maximilians‐University Würzburg Würzburg Germany

**Keywords:** alternative model for personality disorders, loneliness, personality disorders, personality functioning

## Abstract

Loneliness is an aversive state that occurs at elevated rates among individuals with mental health disorders and may reciprocally exacerbate psychopathological processes. Individuals with personality disorders (PDs) appear particularly susceptible; however, knowledge regarding the relationship between loneliness and facets of personality functioning (PF) remains limited. Greater conceptual and empirical clarity is needed to elucidate the mechanisms linking loneliness with maladaptive self‐ and interpersonal functioning and to inform clinical practice. We examined the association between loneliness and PF in a longitudinal cohort of 87 inpatients undergoing psychotherapy programs. Loneliness was measured with the UCLA Loneliness Scale. PF was evaluated with the Semi‐Structured Interview for Personality Functioning and the Level of Personality Functioning Scale. Baseline loneliness scores were positively associated with self‐direction and intimacy, and changes in loneliness were correlated with changes in identity and intimacy. During treatment, significant changes were observed for loneliness and PF, particularly self‐functioning, based on self‐ and clinician ratings. These findings indicate that loneliness is associated with dimensions of self‐ and interpersonal functioning cross‐sectionally and longitudinally. Larger longitudinal studies are needed to elucidate how components of loneliness and PF are interconnected and to identify underlying mechanisms that may inform interventions aimed at reducing loneliness in individuals with PDs.

## Graphical abstract

Loneliness is prevalent in personality disorders (PDs) and linked to impairments in personality functioning (PF). Loneliness was cross‐sectionally associated with PF domains (self‐direction, intimacy), and with changes in PF (identity, intimacy). Ten weeks of psychotherapy significantly reduced loneliness and improved PF, especially self‐functioning. Our findings suggest that loneliness and PF are intertwined, changing concurrently during psychotherapy. Larger longitudinal studies may clarify mechanisms and inform interventions to reduce loneliness in PDs.

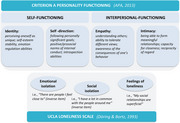

## 1 Introduction

Loneliness constitutes a multifaceted and aversive psychological state that arises from a perceived discrepancy between an individual's desired level of interpersonal connection and the actual quality or quantity of their social relationships [1]. Loneliness and social isolation overlap only to a limited extent [2, 3], indicating that loneliness represents a distinct and predominantly subjective experience. Specifically, feeling lonely poses negative consequences for physical and mental health as well as overall functioning, including depression, cardiovascular disease, and neurodegenerative diseases [4−7].

Although most people experience loneliness at some point [8], individuals with mental health problems, particularly those with personality disorders (PDs), are vulnerable to its more intense and persistent forms [9, 10]. Main evidence on the clinical significance of loneliness in PDs comes from studies in individuals with borderline PD (BPD), indicating that loneliness is related to reduced social functioning [11], and a less positive perception of interpersonal relationships [12], independent of social network size or engagement [11]. However, a meta‐synthesis of 39 studies (*n* = 721) found that across individuals with various PDs or exhibiting PD traits, pervasive feelings of emptiness, disconnection, and alienation were consistently reported [9]. These appear to be linked to experiences of early adversity and further reinforced by later trauma. Developmental models suggest that consequences of adverse childhood experiences often include difficulties in forming secure and satisfying relationships, which may manifest as enduring loneliness and maladaptive schemas of rejection, abandonment, and the perception of a hostile exterior world [9, 13, 14]. These maladaptive schemas may convey persistent beliefs of being unworthy of love or fundamentally disconnected from others, which in turn shape unhelpful interpersonal strategies such as self‐protective withdrawal or maladaptive patterns of emotional regulation that further sustain loneliness [9, 10, 15]. Additionally, while individuals experiencing loneliness frequently express a desire for meaningful and authentic relationships, they may simultaneously exhibit a fear of intimacy and emotional closeness [9, 16, 17]. This paradox suggests that loneliness in PDs reflects not merely a lack of social contact but a disruption in the capacity for relatedness and belonging.

From a differential perspective, research suggested that individuals experiencing persistent loneliness may exhibit personality features that perpetuate their sense of isolation [18]. Within the framework of the five‐factor model of personality, loneliness has been consistently linked to higher levels of neuroticism and lower extraversion [19]. Elevated neuroticism may bias perceptions of the social environment, whereas low extraversion can limit proactive social engagement. The introduction of the DSM‐5's Alternative Model for Personality Disorders (AMPD) [20] and the dimensional concept of ICD‐11 [21] provide new theoretical frameworks to better understand the mechanisms underlying persistent loneliness, and how personality factors contribute to its development and maintenance, particularly in individuals with PDs [22−24]. Both approaches are based on the premise that all PDs share mild, moderate, or severe limitations in self and interpersonal functioning (criterion A) and are characterized by pathological personality traits (criterion B) [20]. *Self‐functioning* comprises impairment in *identity* (perceiving oneself as unique, maintaining stable self‐esteem, and the ability to adequately regulate a spectrum of emotions) and in *self‐direction* (pursuing cohesive and personally significant goals, adhering to positive and prosocial internal norms of conduct, and engaging in introspection). *Interpersonal functioning* encompasses difficulties in *empathy* (understanding and appreciating common motives and experiences of others, being able to tolerate different viewpoints, and being aware of the consequences of one's own behavior on others), and *intimacy* (the ability to form and maintain deep interpersonal relationships, capacity for closeness, and reciprocity of regard) [20]. Criterion B of the AMPD defines pathological personality traits (negative affectivity, detachment, antagonism/dissociality, disinhibition, and psychoticism) to specify individuals’ personal manifestations of personality pathology. This also differs slightly from the ICD‐11's operationalization, which includes an anankastia trait and omits psychoticism [25, 26]. The ICD‐11 explicitly recognizes a sense of alienation or loneliness as a relevant feature within the borderline pattern specifier (ICD‐11 6D11.5) [21], underscoring that loneliness is not merely a byproduct of social isolation but a defining symptomatic difficulty in PD.

In cross‐sectional data, associations of loneliness with the AMPD's criteria A and B have already been observed [27−31]. Particularly, the facets of criterion A, intimacy and self‐direction, were associated with loneliness in our prior study [31]. Intimacy is closely associated with the emotional aspect of loneliness and represents one of the central expectations within ideal social relationships [32]. Dysfunctional self‐direction, characterized by difficulties in goal adjustment or self‐reflection, may impair the ability to formulate new goals for unmet interpersonal needs and to cognitively reappraise experiences of loneliness. In turn, this may exacerbate loneliness, transforming it from a temporary experience into a more persistent condition, as unmet expectations persist due to inadequate coping mechanisms [32]. Identity and empathy were also associated with loneliness, though to a smaller extent [31, 33]. The insufficient development of a personal and social identity is closely associated with loneliness during childhood and adolescence [34, 35]. Additionally, a lack of social identity, particularly when combined with dysfunctional emotion regulation strategies, may contribute to loneliness, especially in individuals with mental health problems [36]. It was further hypothesized that lonely individuals may be less accurate in perceiving the thoughts and feelings of others (i.e., empathy capacities), may fail to accurately recognize appreciation or positive feedback from others, and consequently, such individuals may interpret social encounters as less satisfying or of lower quality [37, 38]. According to self‐reports, the social skills factor of empathy was the strongest predictor for loneliness in 110 adults and university students responding to an ad from a hospital newsletter [37]. However, it remains unclear whether lonely individuals’ tendency to perceive their social skills as poor [38] might lead to inaccurate self‐assessment of empathy, meaning that self‐report questionnaires could misinterpret their actual empathic abilities [37]. Additionally, limited self‐awareness in individuals with more severe impairments in PF may further distort self‐reports of empathy, suggesting that future research should incorporate clinician‐rated measures [39].

Despite growing evidence linking loneliness to dimensions in personality functioning (PF), existing findings are predominantly based on cross‐sectional data, limiting conclusions about stability and temporal dynamics. To this end, we recruited participants diagnosed with PDs (predominantly BPD) or exhibiting varying degrees of impairments in PF within a naturalistic psychotherapy inpatient setting to assess the stability and temporal dynamic of loneliness and PF. Loneliness was assessed with the German UCLA Loneliness Scale (UCLA‐LS) [38, 40], and PF with the German version of the Level of Personality Functioning Scale—Brief Form 2.0 (LPFS‐BF 2.0) [41] as well as the clinician‐rated Semi‐Structured Interview for Personality Functioning DSM‐5 (STiP‐5.1) [42] before and after 10 weeks of psychotherapy. We hypothesized that, beyond baseline associations, improvements in PF would be accompanied by reductions in loneliness across all domains of self‐ and interpersonal functioning, reflecting their conceptual and clinical interdependence. PF was assessed using both self‐ and clinician‐rated instruments, allowing for a more differentiated evaluation of specific domains, such as empathy, that may help explain why some individuals experience persistent loneliness. The overarching aim was to refine theoretical models of the interplay between loneliness and PF in PD and to inform the development of psychological interventions to improve mental health care for this particularly vulnerable population [43].

## 2 Materials and Methods

### 2.1 Participants and Procedure

Participants between the ages of 18 and 65, with sufficient proficiency in German to understand all interviews and questionnaires, were recruited from disorder‐specific, structured 10‐week inpatient psychotherapy programs at the Department of Psychiatry and Psychotherapy of the LMU University Hospital. Participants received either dialectical‐behavioral therapy (DBT) [44] in a primary diagnosis of BPD, or cognitive‐behavioral analysis system of psychotherapy (CBASP) [45] in persistent depressive disorder (PDD). Between June 2018 and July 2024, individuals were screened for eligibility by their attending physicians and provided written informed consent prior to the start of the study.

This study was part of an ongoing naturalistic study of the Department of Psychiatry and Psychotherapy of the LMU University Hospital, LMU Munich, Germany (German Clinical Trial Register ID DRKS00019821), which was approved by the local ethics committee of the Faculty of Medicine, LMU Munich (EK‐No. 713–15). The study was designed in accordance with the Declaration of Helsinki of 1964 as well as its subsequent amendments. All methods followed the German Psychological Society's ethical guidelines, a German adaptation of the “Ethical Principles of Psychologists and Code of Conduct” of the American Psychiatric Association. All participants gave written informed consent.

DBT, as a multicomponent intervention, primarily addresses patients’ self‐harm and suicidal behavior, emphasizing the principal concepts of cognitive‐behavioral therapy integrated with a dialectical approach balancing acceptance and change [46, 47]. In our setting, DBT comprised weekly individual therapy (two 50‐min sessions), and skills training groups (two 100‐min sessions). Additionally, patients participated in weekly occupational therapy (two 75‐min session and one 50‐min session), physical therapy (50 min), a mindfulness group (two 25‐min sessions), art therapy (75 min), and a 1:1 nurse encounter (25 min). CBASP integrates cognitive and behavioral techniques, with a focus on situational analysis and the therapeutic relationship, after identifying individual causal theory conclusions that reflect cognitive and behavioral interpersonal patterns developed through early interactions with significant others [48]. In our program, CBASP included two individual therapy sessions (50 min each), two group therapy sessions (100 min each), and one session (75 min) of the manualized Kiesler Circle Training [49] per week. Additionally, patients received weekly mindfulness training (two 25‐min sessions), occupational therapy (two 75‐min sessions), physical therapy (50 min), art therapy (75 min), and a 1:1 nurse encounter (25 min).

Exclusion criteria included acute suicidality, acute psychosis, current pregnancy, somatically unstable conditions requiring primary medical attention, or a previous stay in the same psychotherapeutic treatment.

At baseline, the Structured Clinical Interview for DSM‐5 (SCID‐5‐CV and ‐PD) [50, 51] was administered by experienced clinical professionals or supervised research assistants. Other clinician‐ratings were also partially conducted by interns who were closely trained and supervised by J.I.K. Interviewers were independent of the clinical teams but were not blinded to the ward or the time of assessment. Baseline data were collected for 120 participants (*n_DBT_* = 52, *n_CBASP_* = 68), with complete data on self‐rated PF and loneliness after treatment available for 87 participants (*n_DBT_* = 36, *n_CBASP_* = 51, study dropout = 11, missing values = 22).

### 2.2 Measures

#### 2.2.1 Loneliness

Loneliness was assessed using the German UCLA‐LS [38, 40]. The UCLA‐LS is a 20‐item self‐report questionnaire with positive and negative connotations of loneliness, rated on 5‐point Likert scales ranging from (1) *not at all* to (5) *totally* [40]. After inverting the positively worded items, the total score and three subscale scores (emotional isolation, social isolation, and feelings of loneliness) correspond to the mean value of their respective items, each ranging from one to five. According to a principal axis factor analysis, the complete solution of 20 items explained 63% of the total variance [40]. The German UCLA‐LS shows good internal consistency (Cronbach's α = 0.89), and acceptable homogeneity (*r* = 0.07–0.77, averaging at *r* = 0.29), consistent with the generally good reliability of the scale across different populations [52]. Cronbach's α was excellent for the total scale before and after treatment (α_pre_ = 0.91, α_post_ = 0.94) and acceptable to excellent for its subscales emotional isolation (α_pre_ = 0.86, α_post_ = 0.89), social isolation (α_pre_ = 0.70, α_post_ = 0.78), and feelings of loneliness (α_pre_ = 0.85, α_post_ = 0.91).

#### 2.2.2 Dimensional Personality Functioning

PF was self‐assessed using the German version of the Level of Personality Functioning Scale—Brief Form 2.0 (LPFS‐BF 2.0) [41] for the preceding 2 weeks. The LPFS‐BF 2.0 comprises 12 items rated on 4‐point Likert scales ranging from (1) *very untrue* to (4) *completely true*, with higher values indicating greater impairment in PF. The sum of the 12 items provides the overall LPFS‐BF 2.0 score, while six items correspond to the two domains of self‐ and interpersonal functioning. The well‐established reliability and convergent validity [53] have been confirmed for the German LPFS‐BF 2.0 for both domains (McDonald's ω ≥ 0.83 for reliability, *r* ≥ 0.72 for convergent validity) [41]. LPFS‐BF 2.0 total scores can be classified as subclinical (≥ 25.9), moderate (≥ 31), severe (≥ 36), and very severe levels of dysfunction (≥ 40.5) [39]. Internal consistency for the LPFS‐BF 2.0 total score was high before and after psychotherapy (α_pre_ = 0.79, α_post_ = 0.88), moderate to high for the self‐functioning domain (α_pre_ = 0.68, α_post_ = 0.83), and acceptable to high for the interpersonal functioning domain (α_pre_ = 0.72, α_post_ = 0.84).

Additionally, PF was evaluated using the clinician‐rated Semi‐Structured Interview for Personality Functioning DSM‐5 (STiP‐5.1) [42]. The interview includes 12 facets rated on a 5‐point severity scale ranging from (0) *no impairment* to (4) *severe impairment*, with higher scores indicating greater impairment. A total PF score and two domain scores—self‐functioning (a combination of identity and self‐direction) and interpersonal functioning (empathy and intimacy)—are calculated as the mean of the respective facet scores. To determine a participant's overall level of impairment, a majority‐based approach was used, that is, if two or more domains were rated (2) *moderate impairment*, the global score was set to moderate according to APA standards [20]. In cases where domain scores differed markedly, the mean of the domain scores was used for the final evaluation. The STiP‐5.1 interviews were conducted by the authors J.I.K. and M.A.R., who received structured training, or by psychology interns who were closely trained and supervised. Studies on the necessary amount of training vary due to the LPFS's theory‐laden concepts [54, 55], but it has been shown that even inexperienced and minimally trained students can effectively apply the STiP‐5.1, achieving acceptable reliability ratings (ICC = 0.51) [56]. All interviewers were independent of the therapeutic teams but were not blinded to the timing of the ratings or the patients’ affiliated wards. The STiP‐5.1 demonstrates high convergent validity (*r* = 0.68−0.78) and interrater reliability (ICC = 0.93, ICC*_range_* = 0.79−0.92), validated for the German version [57], as well as excellent psychometric properties in international samples [58−60]. Cronbach's α was good for the total scale before and after treatment (α_pre_ = 0.80, α_post_ = 0.89) and acceptable to high for its subdomains self‐functioning (α_pre_ = 0.70, α_post_ = 0.86) and interpersonal functioning (α_pre_ = 0.72, α_post_ = 0.80). Internal consistency of the facets scores were lower before treatment (α_pre_ = 0.64 for identity, α_pre_ = 0.55 for self‐direction, α_pre_ = 0.58 for empathy, α_pre_ = 0.64 for intimacy) compared to after treatment (α_post_ = 0.79 for identity, α_post_ = 0.74 for self‐direction, α_post_ = 0.54 for empathy, α_post_ = 0.79 for intimacy), with overall poor scores in empathy.

### 2.3 Statistical Analysis

All statistical analyses were conducted using R version 4.5.0, with a fixed significance threshold of α = 0.05. To examine cross‐sectional associations between loneliness and PF, Pearson correlations were computed between baseline scores on the UCLA‐LS and the LPFS‐BF 2.0 as well as STiP‐5.1, including their respective subdomains (i.e., self‐direction and emotional isolation). Bootstrapping with 5000 iterations was applied to obtain bias‐corrected confidence intervals. To assess longitudinal associations, partial correlations were calculated between change scores (ΔUCLA‐LS and ΔLPFS‐BF 2.0/ΔSTiP‐5.1), adjusting for baseline levels of loneliness and PF. These analyses were conducted for each domain‐specific pairing to account for initial interindividual differences. This approach was used to test the hypothesis that, beyond baseline associations, changes in loneliness and PF are related over time. Changes from baseline to follow‐up in loneliness and PF were examined using paired‐samples *t*‐tests for total scores and subdomains of the UCLA‐LS, LPFS‐BF 2.0, and STiP‐5.1, also employing 5000 bootstrap iterations. All *p*‐values were adjusted according to the false discovery rate (FDR) procedure [61]. Correlation coefficients were interpreted as small (|*r*| = 0.1), moderate (|*r*| = 0.3), and strong (|*r*| 0.5) [62]. Effect sizes for within‐subject comparisons were computed and interpreted using Cohen's *d_z_* and interpreted as small (|0.2|), medium (|0.5|), and large (|0.8|).

## 3 Results

### 3.1 Demographics and Clinical Characteristics

Demographic and clinical characteristics of the sample (*n* = 87, mean age = 35.67 years, *SD* = 13.57; male = 35.6%, female = 62.1%, other = 2.3%) are presented in Table 1. Based on categorical SCID‐5‐PD diagnoses, 56.3% of participants met the criteria for at least one PD, with 42.5% meeting criteria for one PD, 9.2% for two PDs, and 4.6% for three PDs, predominantly including BPD (*n* = 36) or avoidant PD (*n* = 20). According to self‐reported AMPD‐based assessments, 27.6% (*n* = 24) of participants showed mild impairment, 34.5% (*n* = 30) moderate impairment, 16.1% (*n* = 14) severe impairment, and 11.5% (*n* = 10) very severe impairment in PF. Clinician‐ratings based on the AMPD indicated that 22.2% (*n* = 18) of participants exhibited mild impairment, 53.1% (*n* = 43) moderate impairment, 12.3% (*n* = 10) severe impairment, and no participants exhibited very severe impairment in PF.

**TABLE 1:** Demographic and clinical information of the inpatient sample, presented as means and standard deviations, or as frequency and percentage.

Demographic	Total (*n* = 87)
**Age**	35.67 (*SD* = 13.57)
**Male**	31(35.6%)
**Female**	54 (62.1%)
**Other**	2 (2.3%)
**Education in years**	15.31 (*SD* = 3.40)
**Relationship**	
Single/no relationship	49 (56.3%)
Relationship/married	39 (43.7%)
**Occupation**	
No	7 (8.0%)
Yes	38 (43.7%)
Pension	9 (10.3%)
Seeking work	21 (24.1%)
Student	12 (13.8%)
**Living situation**	
Alone	26 (29.9%)
With partner	24 (27.6%)
With parents	16 (18.4%)
Shared apartment	14 (16.1%)
Therapeutic housing	7 (8.0%)
**Personality disorder according to AMPD self rating**	**LPFS‐BF**
No	9 (10.3%)
Mild (subclinical)	24 (27.6%)
Moderate	30 (34.5%)
Severe	14 (16.1%)
Very severe	10 (11.5%)
**Personality disorder according to AMPD clinician‐rating**	**STiP‐5.1 (*n* = 81)**
No	10 (12.3%)
Mild (subclinical)	18 (22.2%)
Moderate	43 (53.1%)
Severe	10 (12.3%)
Very severe	0 (0%)
**Personality disorder according to SCID‐5‐PD**	
Avoidant	20 (23.0%)
Dependent	0 (0.0%)
Obsessive‐compulsive	7 (8.0%)
Paranoid	1 (1.1%)
Schizotypal	0 (0.0%)
Schizoid	1 (1.1%)
Histrionic	0 (0.0%)
Narcissistic	0 (0.0%)
Borderline	36 (41.4%)
Antisocial	0 (0.0%)
**Number of categorical personality disorders according to SCID‐5‐PD**	49 (56.3%)
1	37 (42.5%)
2	8 (9.2%)
3	4 (4.6%)

*Note*: “Male” includes one transgender male, “other” includes nonbinary individuals. Missing information is indicated with the alternative number of participants.

Abbreviations: LPFS‐BF 2.0, Level of Personality Functioning Scale—Brief Form 2.0; *n*, number of participants; SCID‐5‐PD, Structured‐Clinical Interview for DSM‐5 Personality Disorders; *SD*, standard deviation; STiP‐5.1, Semi‐Structured Interview for Personality Functioning.

### 3.2 Associations of Loneliness and PF at Baseline

**FIGURE 1:**
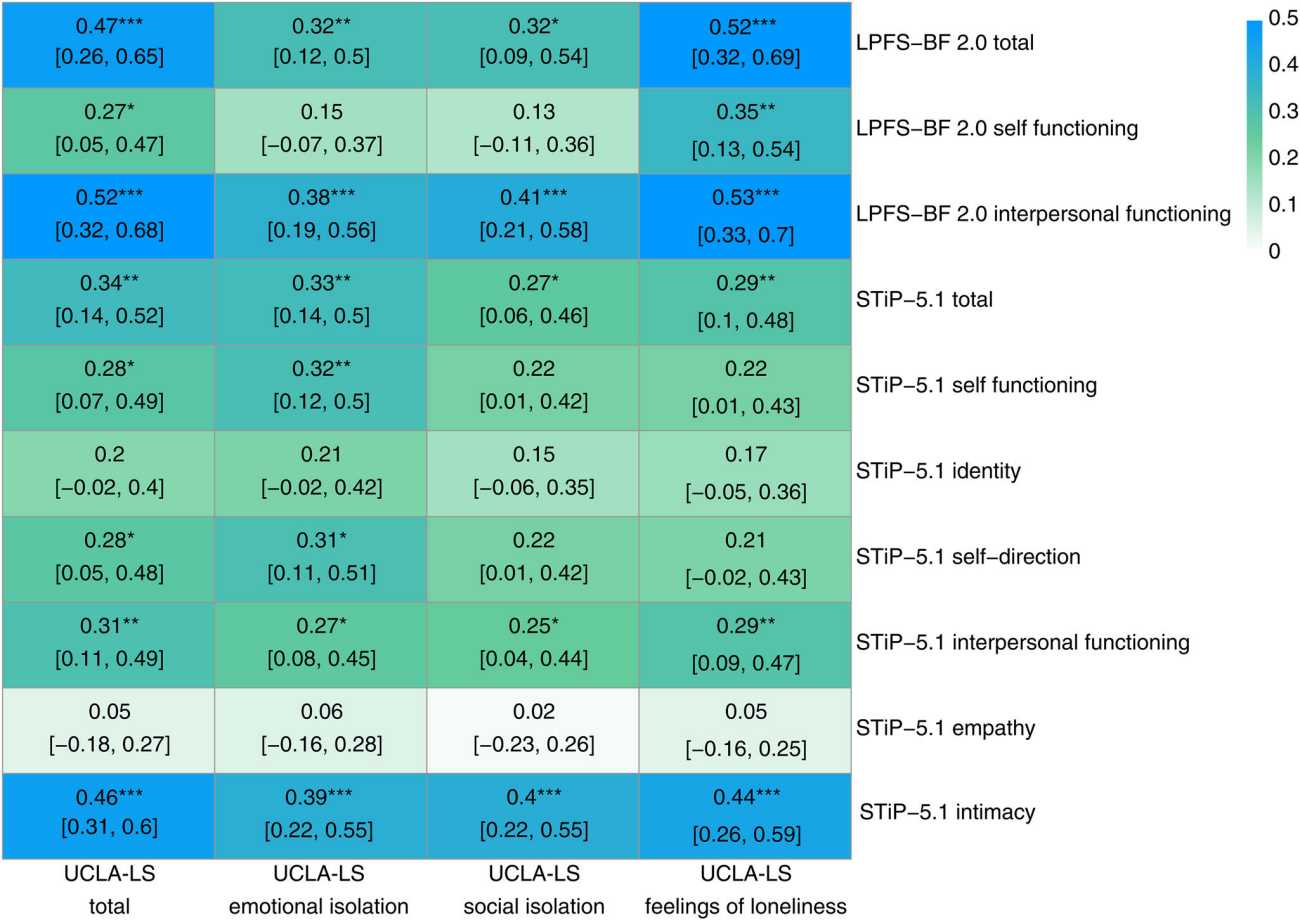
Pearson correlation coefficients for baseline loneliness and impairment in personality functioning with 95% confidence intervals and 5000 bootstrapping iterations. Blue‐shaded correlations indicate strong correlation coefficients, green‐shaded correlations indicate moderate associations, and lightly shaded correlations indicate small to nonsignificant correlations. The more intense the color coding, the stronger the association. FDR corrected *p*‐values with *p < 0.05, **p < 0.01, ***p < 0.001. Abbreviations: LPFS‐BF 2.0, Level of Personality Functioning Scale—Brief Form 2.0; StiP‐5.1, Semi‐Structured Interview for Personality Functioning; UCLA‐LS, UCLA Loneliness Scale.

At baseline, UCLA‐LS total scores were significantly and moderately correlated with both self‐rated (*r* = 0.47, 95% CI [0.26, 0.65], *p_FDR_* < 0.001) and clinician‐rated (*r* = 0.34, 95% CI [0.14, 0.52], *p_FDR_* = 0.004) overall impairment in PF (see Figure 1). Notably, all domains of loneliness showed robust associations with self‐rated interpersonal dysfunction, with the strongest link observed for subjective feelings of loneliness (*r* = 0.53, 95% CI [0.33, 0.70], *p_FDR_* < 0.001), indicating a strong association. In clinician‐ratings, UCLA‐LS total scores showed moderate associations with both self‐ and interpersonal dysfunction. Among the AMPD interpersonal functioning domains, loneliness was strongly associated with impairment in intimacy, whereas no association was observed for empathy. In terms of self‐functioning, loneliness was moderately linked with impairment in self‐direction but not identity.

### 3.3 Changes of Loneliness and PF

A significant reduction in UCLA‐LS total scores was observed after 10 weeks of inpatient psychotherapy (*t*_(86)_ = 2.93, *p_FDR_* = 0.009, *d_z_* = 0.31, 95% CI [0.10, 0.53]), indicating a small reduction. At the subscale level, significant decreases were found in social isolation and feelings of loneliness, whereas emotional isolation remained unchanged (see Table 2). Regarding AMPD‐based PF, self‐rated impairment (LPFS‐BF 2.0) showed a significant strong reduction (*t*_(86)_ = 9.38, *p_FDR_* < 0.001, *d_z_* = 1.00, 95% CI [0.75, 1.26]), indicating improved PF from moderate to subclinical dysfunction [39]. This improvement was evident in both self‐ and interpersonal functioning domains (both *p_FDR_* < 0.001). Clinician‐rated PF (STiP‐5.1) also demonstrated a significant reduction in overall impairment (*t*_(77)_ = 2.81, *p_FDR_* = 0.009, *d_z_* = 0.32, 95% CI [0.09, 0.55]), primarily driven by improvements in self‐functioning (*t*_(77)_ = 3.70, *p_FDR_* < 0.001, *d_z_* = 0.42, 95% CI [0.19, 0.65]). No significant change was observed for interpersonal functioning (*t*_(77)_ = 1.16, *p_FDR_* = 0.329, *d_z_* = 0.13, 95% CI [−0.09, 0.35]). At the facet level, both identity and self‐direction within the self‐functioning domain showed significant small improvements (both *p_FDR_* ≤ 0.009). No significant changes were found in the empathy or intimacy facets (see Table 2).

**TABLE 2:** Results of paired‐samples *t*‐tests on changes in loneliness, self‐ and clinician‐rated personality functioning after 10 weeks of inpatient psychotherapy.

Total sample (*n* = 87)	*M* (*SD*)_pre_	*M* (*SD*)_post_	*MD* (*SD*)_pre‐post_	95% CI	df	*t*‐value	95% *d_z_*	*p*‐value (FDR)
UCLA‐LS total	2.86 (0.73)	2.67 (0.79)	0.19 (0.59)	[0.06, 0.32]	86	2.93	0.31 [0.10, 0.53]	0.009^**^
Emotional isolation	2.48 (0.97)	2.44 (0.99)	0.05 (0.76)	[−0.12, 0.21]	86	0.56	0.06 [−0.15, 0.27]	0.579
Social isolation	3.08 (0.73)	2.88 (0.75)	0.20 (0.63)	[0.06, 0.33]	86	2.93	0.31 [0.10, 0.53]	0.009^**^
Feelings of loneliness	2.93 (0.84)	2.67 (0.90)	0.26 (0.75)	[0.10, 0.42]	86	3.18	0.34 [0.12, 0.56]	0.007^**^
LPFS‐BF 2.0 total	32.49 (6.12)	26.92 (7.38)	5.57 (5.54)	[4.39, 6.76]	86	9.38	1.00 [0.75, 1.26]	< 0.001^***^
LPFS‐BF 2.0 self	18.31 (3.46)	14.98 (4.40)	3.33 (3.75)	[2.53, 4.13]	86	8.30	0.89 [0.64, 1.14]	< 0.001^***^
LPFS‐BF 2.0 interpersonal	14.18 (3.73)	11.94 (3.97)	2.24 (2.58)	[1.69, 2.79]	86	8.09	0.87 [0.62, 1.11]	< 0.001^***^
STiP‐5.1 total (*n* = 78)	1.56 (0.60)	1.35 (0.75)	0.21 (0.66)	[0.06, 0.36]	77	2.81	0.32 [0.09, 0.55]	0.009^**^
STiP‐5.1 self	1.85 (0.67)	1.53 (0.87)	0.32 (0.77)	[0.15, 0.49]	77	3.70	0.42 [0.19, 0.65]	< 0.001^***^
Identity	2.04 (0.70)	1.69 (0.70)	0.35 (0.85)	[0.16, 0.54]	77	3.68	0.42 [0.18, 0.65]	< 0.001^***^
Self‐direction	1.67 (0.88)	1.37 (0.98)	0.29 (0.94)	[0.09, 0.50]	77	2.77	0.31 [0.09, 0.54]	0.009^**^
STiP‐5.1 interpersonal	1.27 (0.70)	1.18 (0.76)	0.10 (0.73)	[−0.07, 0.26]	77	1.16	0.13 [−0.09, 0.35]	0.329
Empathy	1.13 (0.78)	1.03 (0.70)	0.10 (0.82)	[−0.09, 0.29]	77	1.10	0.12 [−0.10, 0.35]	0.329
Intimacy	1.41 (0.85)	1.32 (0.98)	0.10 (0.85)	[−0.10, 0.27]	77	0.94	0.11 [−0.12, 0.33]	0.383

*Note*: UCLA‐LS is divided into three subdomains of emotional and social isolation, and feelings of loneliness. LPFS‐BF 2.0 is divided into two domains (self‐ and interpersonal functioning). StiP‐5.1 is divided into two domains (self‐ and interpersonal functioning) and four facets (identity, self‐direction, empathy, and intimacy).

Abbreviations: CI, 95% confidence interval (after bootstrapping with 5000 iterations); *d_z_*, z‐standardized effect size and 95% confidence intervals; LPFS‐BF 2.0, Level of Personality Functioning Scale—Brief Form 2.0; *M*, mean value; *MD*, mean difference; *n*, number of participants; *p*, FDR corrected *p*‐value; *SD*, standard deviation; StiP‐5.1, Semi‐Structured Interview for Personality Functioning; *t*, *t*‐value with degrees of freedom; UCLA‐LS, UCLA Loneliness Scale.

**p < 0.01, ***p < 0.001.

### 3.4 Associations of Changes in Loneliness and PF After 10 Weeks of Inpatient Psychotherapy

**FIGURE 2:**
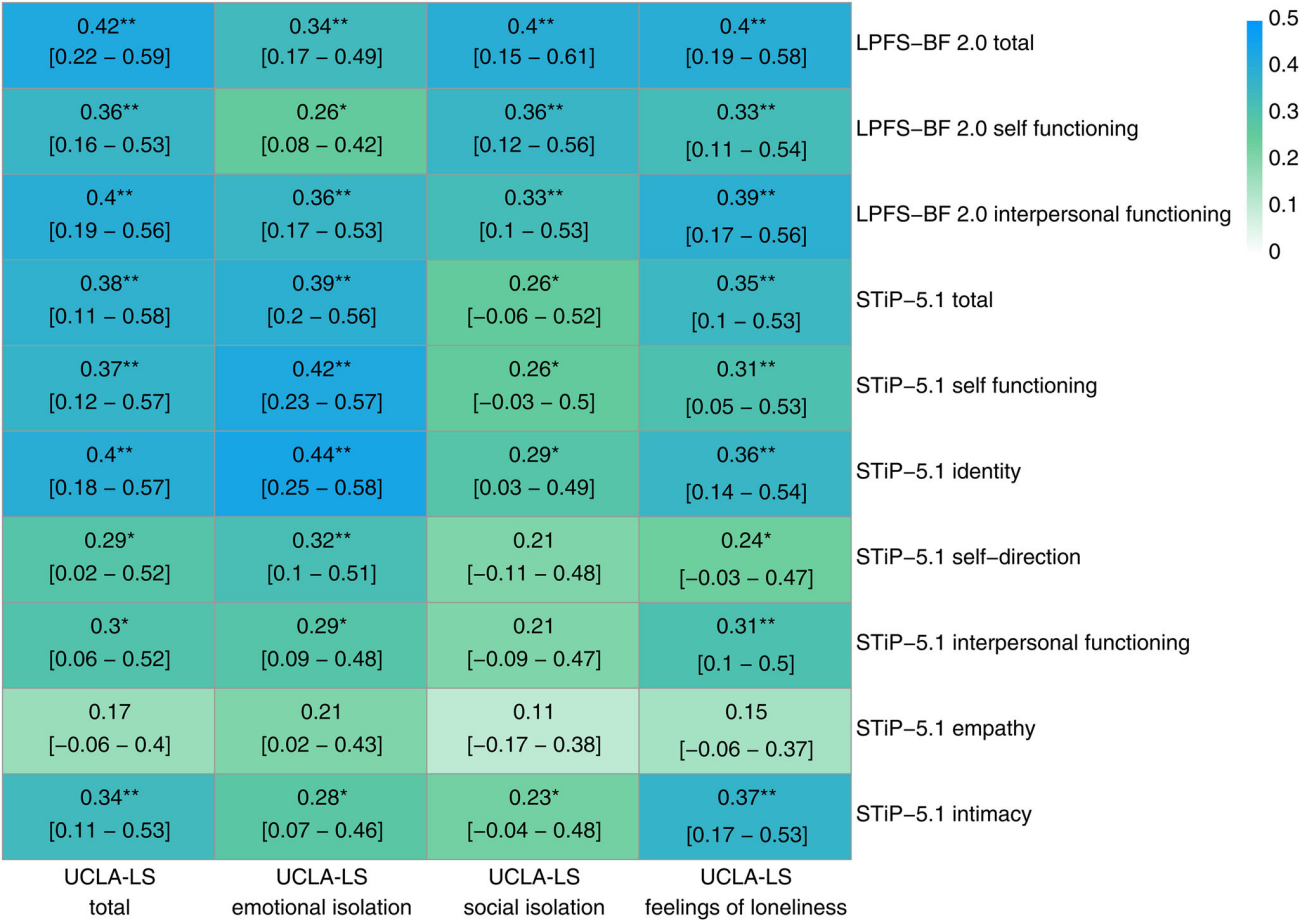
Partial Pearson correlation coefficients for delta loneliness and impairment of personality functioning scores after 10 weeks of disorder‐specific structured inpatient psychotherapy with 95% confidence intervals and 5000 bootstrapping iterations. For each correlation, domain‐specific baseline corrections were conducted to account for initial interindividual differences. Blue‐shaded correlations indicate strong correlation coefficients, green‐shaded correlations indicate moderate associations, and lightly shaded correlations indicate small to nonsignificant correlations. The more intense the color coding, the stronger the association. FDR corrected *p*‐values with *p < 0.05, **p < 0.01, ***p < 0.001. Abbreviations: LPFS‐BF 2.0, Level of Personality Functioning Scale—Brief Form 2.0; StiP‐5.1, Semi‐Structured Interview for Personality Functioning; UCLA‐LS, UCLA Loneliness Scale.

Changes in UCLA‐LS total scores were moderately correlated with changes in self‐rated (*r* = 0.42, 95% CI [0.22, 0.59], *p_FDR_* = 0.001) and clinician‐rated (*r* = 0.38, 95% CI [0.11, 0.58], *p_FDR_* = 0.002) overall personality dysfunction after accounting for interindividual differences at baseline and applying FDR corrections (see Figure 2). Changes in loneliness total and subscale scores were moderately associated with changes in self‐rated self‐ and interpersonal PF. In clinician‐ratings, changes in loneliness scores across all domains were moderately associated with self‐functioning. Among the AMPD self‐functioning domains, changes in UCLA‐LS scores were more strongly associated with changes in identity than self‐direction. Changes in loneliness were also associated with changes in intimacy, but not with changes in empathy.

## 4 Discussion

This cross‐diagnostic longitudinal study investigated associations between self‐reported loneliness and criterion A PF (as defined by the AMPD) in adults participating in two 10‐week disorder‐specific inpatient psychotherapy programs targeting BPD and PDD. PF was assessed using complementary self‐report and clinician‐administered interview measures (LPFS‐BF 2.0; STiP‐5.1). As hypothesized, loneliness and its specific facets were associated with dimensions of PF, both at baseline and with respect to changes observed over the course of the inpatient program. At baseline, loneliness was moderately associated with personality dysfunction, as assessed by both self‐ and clinician‐ratings. These associations were particularly strong for impairments in self‐direction (a self‐functioning domain) and intimacy (an interpersonal functioning domain). Change scores (baseline‐corrected deltas) for loneliness and PF were also moderately correlated, especially with respect to identity and intimacy. Importantly, these changes occurred within the structured environment of 10‐week inpatient psychotherapy programs, during which modest reductions in overall loneliness and substantial improvements in PF were observed, suggesting intertwined change processes. These effects were most pronounced in domains of self‐functioning (i.e., identity and self‐direction), as well as in social isolation, and subjective feelings of loneliness. To our knowledge, this is the first study describing changes in PF, incorporating clinician‐ratings.

In line with previous research, we observed moderate associations between loneliness and both self‐ and interpersonal PF at baseline, particularly with impairment in intimacy and self‐direction [31]. This is in line with the Social Relationship Expectations Framework, suggesting unmet expectations of intimacy as the fundamental core of loneliness [32]. A sense of intimate disconnection is even reflected in UCLA‐LS items such as “I am no longer close to anyone” [63]. Impairments in intimacy might directly hinder the formation of mutually supportive, reciprocal relationships, amplifying the discrepancy between desired and actual relationships. Impairments in self‐direction, in turn, may further contribute to loneliness through more indirect pathways. When individuals struggle to identify meaningful social goals or to re‐evaluate discouraging social interactions (i.e., impairment in self‐direction), loneliness may transform from a transient into a more persistent state, as unmet expectations accumulate without corresponding changes in behavior or coping [31, 32].

Contrasting prior findings, identity and empathy were not significantly associated with loneliness [31, 33, 37]. The lack of association between empathy and loneliness may reflect methodological differences between self‐ and clinician‐rated assessments of empathy. While previous research using self‐report measures has shown that individuals who perceive themselves as less empathetic or socially skilled also feel lonelier [33, 37], clinician‐rated empathy in the present study was unrelated to loneliness. This discrepancy suggests that loneliness may be more closely linked to subjective perceptions of empathic abilities. Lonely individuals may possess adequate empathy behaviorally but maintain negative or distorted self‐perceptions of their social abilities, which could perpetuate loneliness. However, this interpretation remains hypothetical, as the LPFS‐BF 2.0 only distinguishes between self‐ and interpersonal PF.

A second major finding of our study was the parallel change in loneliness and PF observed during the intensive 10‐week inpatient psychotherapy programs for individuals with BPD and PDD. Interestingly, the strong improvements in PF align with previous research demonstrating the sensitivity of PF measures to psychotherapy, particularly within treatment durations ranging from 8 weeks to 94 days across studies [64−66]. This represents a relatively short timeframe, especially when contrasted with the traditional view of personality pathology as stable and resistant to change [20]. Both DBT and CBASP are designed to target core components of PF, such as emotion regulation, self‐reflection, and interpersonal effectiveness, which are increasingly recognized as modifiable targets [67−74]. Evidence from these and similar inpatient programs suggests that even relatively brief interventions can yield measurable improvements in PF, potentially serving as a catalyst for longer‐term personality development and stabilization. In a previous study, the self‐functioning domain of the LPFS‐BF 2.0 appeared to be particularly sensitive to change following specialized treatment for PDs with an average duration of 92.13 days (*d* = 1.22) [65]. In contrast, in the present study, no difference in sensitivity to change was observed between self‐ and interpersonal PF after 10 weeks of treatment. Our interpretation of these changes remains limited to the observed associations between loneliness and PF domains within this structured and interaction‐rich therapeutic environment.

This improvement was accompanied by statistically significant, albeit small, changes in clinician‐rated total and self‐functioning, whereas no significant change was observed in interpersonal functioning. To our knowledge, this is the first study to report PF changes assessed with the STiP‐5.1 in a psychiatric treatment setting; notably, these changes appeared more modest than those observed in self‐report measures. Beyond the possibility of a Type II error due to the moderate sample size, self‐reports may be more sensitive to subtle changes (e.g., shifts in self‐perception or increased self‐awareness), whereas nonblinded clinician‐ratings may have relied more on observable or relatively stable markers [75]. Moreover, self‐report measures of PF and loneliness may be more strongly influenced by state‐dependent factors such as increased mood [76, 77], hope and motivation during treatment, or the supportive structure of the inpatient setting. These contextual and affective changes may temporarily enhance one's perception of PF and social connectedness, contributing to greater observed change in self‐report measures relative to clinician‐ratings. In contrast, clinician‐rated PF may have reflected more enduring aspects of PF that are less dependent on dynamic affective or structural states, resulting in comparatively smaller, yet more accurate, change scores. It should also be noted that the 10‐week treatment period may not be sufficient to capture substantial changes in PF, that is, identity, self‐direction, intimacy, and empathy. Detecting substantial changes may require the accumulation of new experiences, which extend beyond the timeframe of the inpatient setting. Hence, the more modest changes observed in clinician‐ratings might reflect both the short timeframe and the stability of the constructs, whereas self‐reports could be capturing earlier, more state‐dependent indicators of therapeutic progress. Consequently, this differential pattern may reflect differences in measurement sensitivity as well as the temporal dynamics of change in PF.

Across the 10‐week psychotherapeutic treatment period, baseline‐corrected changes in loneliness were moderately associated with both self‐ and interpersonal PF according to self‐ and clinician‐ratings. Evidence on such dynamic associations is scarce. Previous work has shown that intimacy and identity were moderately related to loneliness in adults seeking mental health services, with weaker associations for self‐direction and empathy [33]. To date, however, no study has examined whether changes in loneliness and PF co‐occur during psychotherapy. The present findings underscore the importance of PF in the identity domain, that is, the capacity to recognize oneself as unique, maintain stable self‐esteem, and effectively manage a wide range of emotions. The reduction of loneliness was substantially associated with changes in this domain, although identity was not associated with loneliness at baseline, contrasting previous findings [33, 36]. This is in line with previous research suggesting that identity and loneliness influence each other over time [34]. As identity stabilizes in therapy (e.g., through the development of a clearer, cohesive self‐concept and improved emotion regulation strategies), individuals may engage more in reciprocal relationships, thereby reducing loneliness. In other words, higher levels of loneliness may hinder in‐depth self‐exploration and forming new commitments, whereas increasing certainty about one's identity may facilitate closer and more meaningful connections with others, thus alleviating loneliness [34]. However, beyond structured therapeutic modules, ward interactions may also contribute to reduced loneliness by fostering a sense of belonging, which in turn stabilizes self‐esteem and reduces reliance on maladaptive regulation strategies. Further, looking at cross‐sectional associations, the level of identity impairment does not necessarily determine how lonely someone feels, a pattern that has been noted in earlier research reporting only small effect sizes [33].

Additionally, changes in intimacy and self‐direction were consistently linked to changes in loneliness, with small to moderate effect sizes, whereas empathy showed no significant association. Empathy enables individuals to understand others, tolerating different viewpoints, and being aware of the consequences of one's own behavior [20], yet it may not guarantee reciprocal or emotionally satisfying relationships. This pattern suggests that loneliness extends beyond the scope of empathy and is more closely tied to difficulties in building close reciprocal bonds (intimacy) and pursuing cohesive goals and adaptive self‐reflection (self‐direction). The lack of association may likewise be explained by methodological differences between self‐ and clinician‐rated assessments of empathy. While cross‐sectional studies using self‐report measures have linked higher empathy with lower loneliness [37], the present findings suggest that subjective improvements in empathy, rather than interview‐based changes in impaired empathy, may be more relevant for reductions in loneliness. However, as no previous studies have examined parallel changes in empathy and loneliness, including those based on self‐ratings, this interpretation remains highly tentative.

In sum, the present findings indicate that loneliness and PF are closely intertwined, both at baseline and across their trajectories over time. This dynamic association supports the view that loneliness is not merely a consequence of reduced social contact or an isolated interpersonal issue but rather may be rooted in multiple domains of PF, particularly identity, in individuals with BPD and PDD who appear especially vulnerable. One possible explanation is that impairments in self‐ and interpersonal functioning, such as difficulties in maintaining a coherent identity, setting meaningful goals, or forming reciprocal relationships, may amplify the perceived gap between desired and actual social connectedness, thereby reinforcing loneliness. Persistent loneliness may, however, impede opportunities for meaningful engagement, undermine self‐esteem, and limit experiences that foster adaptive self‐ and interpersonal functioning, thus creating a feedback loop between PF and loneliness over time. The robustness of these associations across baseline and treatment‐related change underscores their consistency over time and highlights shared mechanisms that warrant further investigation.

### 4.1 Limitations

While we believe our findings are noteworthy, we acknowledge substantial limitations: (1) The relatively small sample size hampers the generalizability of our findings and limits statistical power. (2) For a longitudinal study, the observation period was very short, with only two time points of measurement, and the treatment setting was artificially structured and inherently hyper‐social, and, importantly, lacked a control group for comparison. Therefore, the observed changes should not be interpreted as specific effects of the psychotherapeutic intervention, nor as indicative of changes that would naturally occur in participants’ everyday social environments over time. (3) We recruited participants from two disorder‐specific inpatient psychotherapy programs—DBT for BPD and CBASP for PDD, that exhibited relatively low levels of categorical personality pathology compared to expectations based on these diagnostic profiles [78, 79]. Further, in our sample, 37.9% of participants based on self‐ratings and 34.6% based on clinician‐ratings did not meet the threshold for a PD (i.e., exhibited subclinical or no impairment in PF), and it remains uncertain whether these individuals would meet diagnostic criteria for a PD according to dimensional assessments. This may reflect the selective nature of both programs, which require a certain level of stability for participation and may thus limit the generalizability of our findings. (4) A further limitation concerns the interpretation of discrepancies between self‐rated and clinician‐rated PF change. Although the dual assessment is a strength of this study, we did not examine whether changes in self‐reported PF and loneliness were influenced by state‐related factors such as mood (i.e., changes in depressive symptoms). Self‐reported PF improvements, therefore, may partly reflect temporary reductions in distress during inpatient treatment rather than enduring changes in PF. (5) Clinician‐rated STiP‐5.1 interviews were conducted by different, nonblinded raters, which may have influenced post‐treatment evaluations to some extent, and the internal consistency of the STiP‐5.1 facet scores was lower at baseline. While the exact cause remains unclear, the higher Cronbach's α at post‐treatment may reflect increased patient self‐reflection or stability in self‐concepts resulting from psychotherapy. Alternatively, it could indicate lower rater precision at baseline. (6) The observed changes in loneliness should also be interpreted with caution, since we did not assess possible confounding variables, that is, whether patients experienced interactions with peers and caregivers as supportive, whether interpersonal differences influenced the frequency of contact, or whether reductions in loneliness were sustained following discharge. (7) Lastly, interpretation of the UCLA‐LS subdomains is limited by the lack of consensus on its factor structure and theoretical framework—either distinguishing between emotional and social loneliness, or separating loneliness from isolation [40, 80, 81]—underscoring the need for further psychometric clarification.

### 4.2 Implications

Our findings suggest that the previously reported associations between loneliness and PF persist over time, while both constructs showed significant improvements following relatively short but structured, intensive psychotherapy treatments. However, an important open question concerns the nature of this relationship: (1) if loneliness is essentially an experiential expression of reduced PF, one would expect strong concurrent associations at each time point; (2) if loneliness is rather a consequence of impaired PF, baseline PF should prospectively predict changes in loneliness during treatment; and (3) if loneliness amplifies or maintains impairments in PF, baseline loneliness should predict treatment‐related changes in PF, and changes in PF could in turn mediate improvements in loneliness. Future studies with larger samples and repeated assessments could apply cross‐lagged panel models and mediation analyses to further test these possibilities. Given the growing relevance of dimensional PD models, research should include and investigate individuals with various degrees of PF severity, since studies on loneliness and personality pathology mainly focus on categorical BPD [10]. Changes in PF and loneliness should also be considered in interaction with criterion B traits, particularly negative affectivity and detachment [82, 83], including both AMPD and ICD‐11 LPF trait measures [84]. Additionally, the discrepancy between self‐ and clinician‐rated change underscores that both perspectives provide relevant information, and that future studies should incorporate and systemically compare both assessment modalities while accounting for mood‐related state factors to obtain a more comprehensive understanding of change over time.

To date, no interventions have been developed that specifically target loneliness in PD. However, interventions targeting AMPD criteria A and B may indirectly reduce loneliness by targeting shared and proposed underlying mechanisms [43]. Loneliness is conceptualized as involving maladaptive social perceptions, cognitions, emotions, behaviors, physiological responses, and diminished social connectedness, which together may perpetuate loneliness at both intra‐ and interpersonal levels. Strategies proposed by our group may selectively enhance specific domains of PF and thereby reduce loneliness, although a validated integrative model of these interactions is still lacking [43]. Accordingly, a deeper understanding of the mechanisms underlying the interplay between loneliness and PF is urgently needed to inform the development of effective psychological interventions for individuals with PDs, who are at heightened risk for persistent loneliness. Explicit assessment of loneliness early in psychotherapy may help clinicians identify individuals who are particularly vulnerable and at increased risk of PD‐related symptomatology due to impairments in both intrapersonal and interpersonal functioning (i.e., in self‐direction and intimacy) [9, 10, 85]. These individuals may benefit most from interventions that explicitly address loneliness in conjunction with strategies aimed at improving PF in identity and intimacy domains.

## 5 Conclusion

Loneliness and impairments across domains of PF were significantly associated both at baseline and in terms of baseline‐adjusted change scores, following the 10‐week structured, inpatient psychotherapy programs. These findings support the view that loneliness is not merely a consequence of reduced social contact or an isolated interpersonal difficulty but may instead be deeply embedded across multiple domains of PF. This appears particularly salient for individuals with BPD and PDD, who may be especially vulnerable to persistent loneliness. Future research should examine the directionality and underlying mechanisms of these changes, using broader dimensional frameworks, such as the AMPD and ICD‐11, within transdiagnostic designs. Such approaches may better capture the complexity of loneliness in the context of personality pathology and inform the development of targeted, mechanism‐based psychological interventions.

## Author Contributions

J.I.K. and M.A.R.: Conceptualization, data curation, formal analysis, investigation, methodology, writing – original draft, writing – review and editing; B.B.B.: Conceptualization, data curation, investigation, writing – review and editing; N.W.: Conceptualization, data curation, writing – review and editing; J.W. and K.M.: Data curation, writing – review and editing; K.B., A.J., R.M., and F.P.: Conceptualization, writing – review and editing.

## Conflicts of Interest

F.P. is a member of the European Scientific Advisory Board of Brainsway Inc., Jerusalem, Israel, and the International Scientific Advisory Board of Sooma, Helsinki, Finland. He has received speaker's honoraria from Mag&More GmbH and the neuroCare Group. His lab has received support with equipment from neuroConn GmbH, Ilmenau, Germany, and Mag&More GmbH and Brainsway Inc., Jerusalem, Israel. R.M. has received financial research support from the EU (H2020 No. 754740) and served as PI in clinical trials from Abide Therapeutics, Böhringer‐Ingelheim, Emalex Biosciences, Lundbeck GmbH, Nuvelution TS Pharma Inc., Oryzon, Otsuka Pharmaceuticals, and Therapix Biosciences. The remaining authors declare that the research was conducted in the absence of any commercial or financial relationships that could be construed as a potential conflict of interest.

## Data Availability

The data that support the findings of this study are available on request from the corresponding author. The data are not publicly available due to privacy or ethical restrictions. Analysis code and a detailed data dictionary are available via the Open Science Framework (OSF).
